# Contextual Factors Influencing Young Athletes’ Decision to Do Physical Activity and Choose a Sports’ Club: The Case of Portugal

**DOI:** 10.3390/healthcare10020347

**Published:** 2022-02-11

**Authors:** Gonçalo Santinha, Rafaela Oliveira, Luís Jorge Gonçalves

**Affiliations:** 1 GOVCOPP, Department of Social, Political and Territorial Sciences, University of Aveiro, 3810-193 Aveiro, Portugal; 2 Department of Social, Political and Territorial Sciences, University of Aveiro, 3810-193 Aveiro, Portugal; rafaelaoliveira@ua.pt; 3 Águeda School of Technology and Management (ESTGA-UA), University of Aveiro, 3750-127 Águeda, Portugal; luisjorge@ua.pt

**Keywords:** sports practice, sports clubs, basketball, young athletes, decision, influence factors, catchment area

## Abstract

Physical activity and sports are a central part of individuals’ lives throughout the life cycle. During adolescence, its regular practice may contribute to the development of healthy adult lifestyles, decreasing chronic disease incidence. Therefore, the reasons that drive adolescents to start practicing sports in a certain club may be multiple and understanding such causes can be important to design and implement public policies to promote active lifestyles for everyone. In this article, we report the core findings of a research on why young athletes do sports, how they choose their team-training club and how COVID-19 has had an impact on their routines. From a methodological viewpoint, a questionnaire was developed and sent to sport clubs located in NUTS2 Centro Region, Portugal, and results were analyzed through the use of geographic information systems and statistical analysis, namely association tests (Chi-square test), difference tests (Mann–Whitney test and Kruskal–Wallis test), logistic regression and descriptive analysis. Findings show that family, age group, friends, proximity to sports facilities, teammates, and club conditions are the factors that influence adolescents the most. In short, external factors have a significant preponderance to practice physical activity and choose a sports’ club. These findings can provide useful insights for clubs, coaches and policy-makers to become more aware of the relationship between sports practice and accessibility to sports halls, as well as the athletes’ behavior and their connection with the club and basketball practice. As such, these findings can motivate the design of initiatives and strategies to boost sports practice and to find ways for clubs to attract more adolescents.

## 1. Introduction

Back in the 1950s, Morris and colleagues’ [1] seminal work showed epidemiologic evidence between physical activity and cardiovascular diseases. Since then, accumulated evidence has shown that physical activity has potential positive impacts on citizens’ health, both in terms of prevention (lower incidence rates of coronary heart disease, type 2 diabetes mellitus, hypertension, some cancers and osteoporosis) and in the treatment of several chronic diseases [2,3,4].

These days, physical activity and sports are a central part of individuals’ lives throughout the life cycle, from childhood to old age. During adolescence, in which physical activity is commonly higher, its regular practice may contribute to the development of healthy adult lifestyles, decreasing chronic disease incidence [3,5]. The reasons that drive adolescents to start practicing sports may be multiple and understanding such causes can be important to design and implement public policies to promote active lifestyles for everyone [6,7].

The sports environment is complex, composed of a variety of agents and factors, but it is intended to provide multiple benefits to individuals [8]. For instance, sports club activities may form a highly motivating health promotion context for youth [9]. This sector ranges from informal physical activity to high performance sports. Sports clubs are basic units of sport, which are the main productive entities of the sector [10]. According to the national law of sports and physical activity [11], sports clubs are “legal entities of private law, constituted as a non-profit association, whose scope is the promotion and direct practice of sports”.

Although the practice of informal physical activities is beneficial to individuals, the participation in organized sports brings additional benefits. According to Hardy [12], for instance, participating in organized sports allows individuals to acquire new skills, interact with other people, face and overcome challenges, and work as a team. This reveals the pivotal role that sports clubs play in the growth and development of young people. As such, clubs are paramount for young people, both in teaching the sport and in mediating values. In turn, Gomes [13] states that young people join sport clubs either willingly or through the influence of other agents and, as such, they can withdraw at any time. However, sport club managers and coaches are not fully aware of the reasons young people join their own team club and what drives them to stay there. The importance of motivating young people to practice sports activities is widely recognized in literature, and joining sports clubs is quite relevant. Group activities are more popular than individual ones, as they have a strong social support, providing in turn a strong development of satisfaction, group affiliation and commitment to the individual and to others [14]. It also allows young people to more easily become aware of their development and improvements in physical condition.

Studies on this theme, as opposed to those ones focusing on factors that drive young people to start practicing physical activity or sports in general, are limited. This study fills this gap by identifying how and why adolescents choose their team-training clubs. While true that adolescents are a quite heterogeneous group, their expectations and preferences of different groups of adolescents are understandably different [15], and the reasons that induce them to choose a certain sport or club may differ from each other. However, certain contexts may be more conducive to the practice of certain activities or may influence adolescents’ choice [16]. We hypothesize that the neighborhood environment and the proximity to the sports club, and social factors work jointly to account for adolescents’ decision. On the other hand, the COVID-19 pandemic has had a great impact in several areas of society, including physical activity and sports [17]. It is therefore important to understand the impacts of this public health phenomenon in sports organizations and in sports routines of individuals.

The main purpose of this study is to discern why young basketball players do sports, how they choose their team-training club and how COVID-19 has had an impact on their routine. Using a quantitative methodological approach, this study was conducted in a specific territorial context from a southern European country: Aveiro District, Portugal. Portugal has a great relationship with sports, being considered an exporter of talents and technical knowledge in various modalities. Apart from football, basketball is also well grounded in Portugal, with internationally recognized protagonists such as Ticha Penicheiro and more recently Neemias Queta now playing in the NBA. With several clubs playing in the first and second national leagues, it is usual for adolescents to play basketball in the region of Aveiro, for leisure or competition.

The remainder of this article is structured as follows. The next section summarizes the theoretical background of the main factors pointed out in the literature that influence the practice of physical activity and sports. Due to the impact caused by the present pandemic phenomenon in society, the subsequent section briefly states how the practice of physical activity and sports may have changed during the COVID-19 pandemic. The fourth section focuses on the empirical case: the methodology and results are presented, in which data were analyzed according to gender, age group, and clubs. The last two sections discuss the findings obtained and present some final remarks on the issue at stake.

## 2. Factors That Influence the Practice of Physical Activity and Sports

Physical activity and sports allow individuals to have a healthier lifestyle and a better quality of life [14,18], both terms being defined by Caspersen et al. [19] (p. 126), as “any bodily movement produced by skeletal muscles that results in energy expenditure”. But even though these designations share similarities, such as being related to activities performed by individuals in their daily life, they differ in some important aspects. Exercise is a subcategory of physical activity; it is planned, structured and repetitive [17,19,20,21]. Sports, on the other hand, are even a more specific form of physical activity; they are structured, competitive, subject to rules and strategies [14,20,21].

Usually, it is during adolescence that a healthier life path becomes a choice [22]. During this phase, young people form their identity and are also more vulnerable to external influences [22], which means that when physical/sporting activity is encouraged, they are more likely to perpetuate this routine into their adult life. The consequences of this habit are rather positive [14,23], since the different experiences that promote a development of individual, social and relational skills also provide a better academic, educational and emotion management, significant to the integral development of someone’s personality [14].

In this context, understanding the way decisions are made can be an important step towards the interpretation of young people’s behaviors. Decision-making involves making a choice amongst alternative courses of action, ultimately indicating what is intended by the person who made the decision [20]. Individuals can choose to be active or not, and can choose if they are willing to engage in physical activity and sports. The choice of the sports’ modality indicates their interests, motivations and their own characteristics, but it is also a complex process, as the decision may result from their own will, their skills or pressures from the environment. For example, in the study mentioned by Coakley and White [24], young people reveal that the decision to practice sports activities is related to relationships with friends, material resources and contextual influences.

In this sense, some factors may influence the choice of a healthier lifestyle during adolescence. Firstly, because demographic-biological factors determine that sport practice tends to decrease with age [6,14]. Moreover, although girls are becoming increasingly sporty, boys still tend to be more active [6,21,24]. Secondly, those who were born in high socioeconomic status families are more likely to practice sports [21,24,25]. Thirdly, sociocultural factors, such as family, friends, physical education teachers and coaches, have an impact on young individuals, encouraging them to choose certain modalities, sport clubs and routines [6,14,21]. Moreover, environmental factors (e.g., availability and proximity of sports facilities) are also important, not only in terms of perpetuating the habit, but also in terms of choosing the type of sport and the club [14,26,27]. Lastly, the characteristics of the sport and of the individuals can also promote or hinder sports practice adherence [21,28].

In short, there are several issues to consider when analyzing why, how, and what makes young people choose certain sport/physical activity paths. They may be connected to social issues, pleasure, and power of choice and sense of well-being [20].

## 3. The Practice of Physical Activity and Sports during COVID-19 Pandemic

The COVID-19 pandemic has forced societal changes, based on thorough measures of individual and communitarian protection, ranging from compulsory social distancing to quarantine, which have resulted in major changes in both work and social lives [17,24]. As Adam and colleagues [29] point out, the world was forced to reinvent himself and people acquired new behaviors, including the practice of physical activity at home.

For sport federations and clubs, the pandemic meant a reduction in terms of income and socialization, as the ending of sport competitions was imposed and space restrictions during classes became mandatory [30,31]. On top of that, the increasing needs of hygiene, the training limitations, lack of facilities for training and lack of competition in training levels, translated in a reduction of athletes due to dropout and increased costs [30,32].

Many athletes drooped out because of competition stoppages, demotivation, or fear of being infected, since there was a risk of putting teammates and families at stake [29]. Nevertheless, some of them underwent a change in the way they got involved in sports, being forced to train at home, using digital platforms or resorting to neighborhood outdoor spaces and parks close to home [24,30].

However, those who did it were still susceptible to situations of anxiety, frustration, and sleep disorders, putting their well-being in jeopardy [29]. Such portrait shows how important is to maintain physical/sports activity, as its practice benefits individuals, both physically and mentally [17,31].

## 4. Materials and Methods

### 4.1. Participants and Procedure

Data was collected from February to April 2021 through an online survey in Google Forms, which was shared via institutional contacts, namely by the Aveiro Basketball Association. The survey was sent to all female and male athletes of two different age groups (10–12 years and 13–14 years) of the 25 clubs playing in Aveiro District (*n* = 587), located in NUTSII Centro Region of Portugal. As not all athletes from all sports’ clubs were willing to collaborate, the sample was quite low for some clubs. The ones that did not have a representative sample in both age groups were therefore excluded from the study.

**Figure 1:**
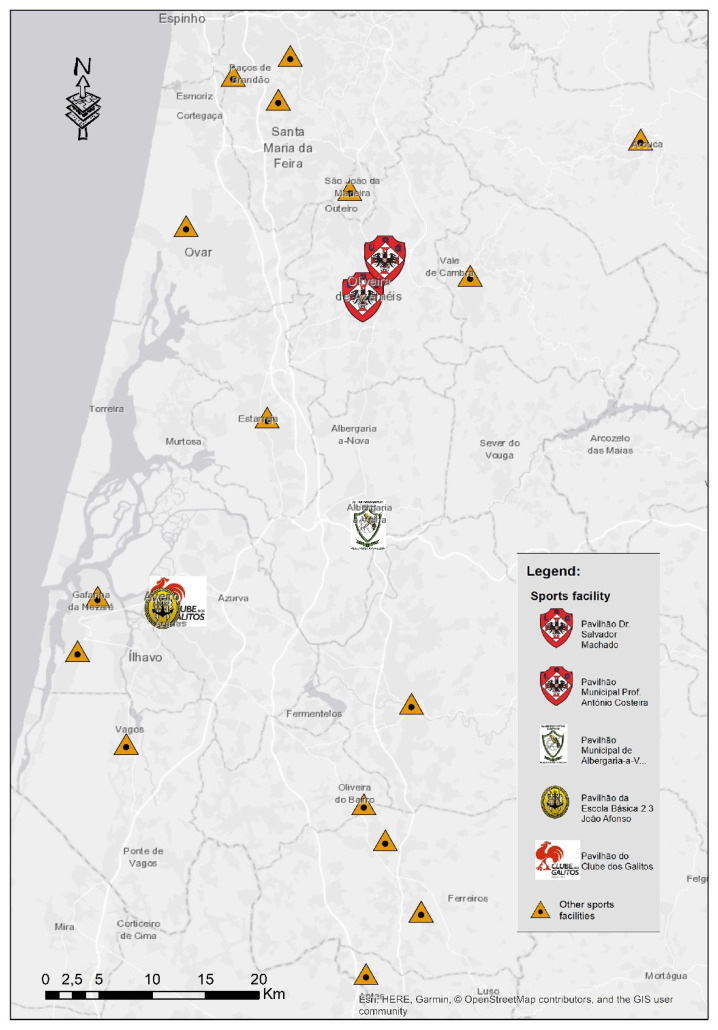
Location of clubs’ infrastructures (Aveiro District).

One hundred and one replies were considered valid for this study, 66.3% men (*n* = 67) and 33.7% women (*n* = 34). The sample includes 47 athletes with 10–12 years old (46.5%) and 54 with 13–14 years old (53.5%). The participants considered for the study were part of four different basketball clubs: 23 (22.8%) of Clube Desportivo do Campinho, 39 (38.6%) of Clube dos Galitos, 15 (14.9%) of Sport Clube Beira-Mar and 24 (23.8%) of União Desportiva Oliveirense. Figure 1 maps the basketball clubs located in Aveiro District.

For more than half of the participants (54.5%, *n* = 55), basketball was the first sport they played and for the majority (55.4%, *n* = 56) external factors (parents’ wishes; friends’ influence) were the main reason for choosing this sport. On the contrary, for 44.6% of the athletes (*n* = 45) internal factors, such as the taste for the sport and for team sports, were mentioned as important ones. The years of practice of the sport vary between 1 and 10 years. The mean is 3.5 years, and the standard deviation is 1.98. Some of these athletes (13.9%, *n* = 14) have already practiced the sport in other clubs before coming to the current one.

### 4.2. Questionnaire

The questionnaire was developed by the authors based on the (scarce) existing literature on the issue at stake, as referred to in the previous sections. A pilot-test was carried out with the Aveiro Basketball Association, whose inputs were included in the final script of the questionnaire. An online link of the questionnaire was then sent by the Aveiro Basketball Association to all clubs by email and included an informed consent describing the study, the aim and topics involved and informing participants about the confidentiality of their answers.

Through this instrument, we collected data regarding: (i) sociodemographic characteristics (gender, age, number of brothers, postal code, level of training and sports club); (ii) what influenced athletes’ decisions regarding sports practice (5 items) and the reasons that led them to choose the sport modality and the club (17 items); (iii) the contexts in which athletes were involved, through 13 items related to family, friends, coaches, and teammates, and 6 items related to the physical environment (proximity and accessibility to sports facilities); (iv) the changes that COVID-19 pandemic caused to athletes’ routine, with 7 items related to physical activity during this period and 4 regarding the impacts they felt. With these data, we were able to collect information regarding individual internal factors for playing basketball (to like practicing sports in general and/or to have the opportunity to experience new challenges), external factors (the influence of friends, relatives, parent’s linkage with the sports club, teammates and/or coaches in making decisions regarding basketball) and contextual factors (the importance of the sports facility’s location, the conditions of sports clubs (physical, material and number of athletes) and/or the distance between the area of residence and the sports facility).

The questionnaire encompassed closed-ended questions. Some questions comprised a Likert scale ranging from 1 to 5, in which, depending on the question, 1 meant “strongly disagree”; “very bad” or “not at all satisfied” and 5 meant “strongly agree”; “very good” or “very satisfied”, respectively.

### 4.3. Data Analysis

Data statistical analysis was conducted using SPSS (version 27.0). A descriptive analysis of the athletes’ answers was performed using measures of central tendency (mode and median), for the nominal and ordinal variables, and of dispersion (Frequency and Interquartile range). Chi-square and Mann–Whitney tests were used to explore significant associations or differences between gender and age group, the latter being a non-parametric test applied to two independent samples [33]. Chi-square and Kruskal–Wallis tests were also used to determine if the same associations or differences exist between clubs. The Kruskal–Wallis test is a non-parametric method used to compare more than two independent samples of equal or different sizes [33]. These analyses were applied to questions, but in some cases the results were not statistically valid. The most reported factors were then inserted into logistic regression models to identify the predictors that influence the decision of athletes when choosing a sports club. For each model, the dependent variable was the club under analysis and the independent variables were gender, age group, family financial conditions, friends, teammates, coach and club conditions. These variables are dichotomous. Gender, age group, and the importance of teammates take on a value of 0 if “no” and a value of 1 if “yes”. The remaining variables, initially following a Likert scale (1 to 5), were transformed into dichotomous variables as well, taking the value of 1 in case of “have importance for the choice of club” (assigned values 4 and 5 in the Likert scale) and 0 in the case of “have no importance for the choice of club” (assigned values 1, 2 and 3 in the Likert scale). Finally, the significance level considered was 0.05.

In addition, using Geographic Information Systems technologies, specifically the opensource software QGIS, clubs’ infrastructures and athletes’ households’ location were mapped to determine if athletes tend to live near or far from their club. The address of the sports facilities was collected from the official municipal websites, as well as from Google Maps. The home addresses of the survey athletes and those of the sports facilities were geocoded using the geocoding process, via the OpenRouteService API (© openrouteservice.org by HeiGIT | Map data © OpenStreetMap contributors).

## 5. Results

### 5.1. Gender Differences and Associations

The presence of a known teammate or coach was an important factor for both genders for electing a club, with 67.2% of the boys knowing up to 5 people and 55.9% of the girls knowing even more than 5 people. On the other hand, the accessibility factor (indicated by the distance between the athletes’ household and the sports facilities) was more important for boys than for girls (U = 891.50; *p* = 0.065).

Regarding the factors that may have influenced the reasons for choosing a club, girls gave more importance to the material conditions of the club (U = 715.00; *p* = 0.002) and the performance of their teams (U = 665.00; *p* ≤ 0.001) than boys. The girls, compared to the boys, manifest more that in the club where they play, they have all the conditions to achieve the goals they want as basketball players, U = 764.00, *p* = 0.004. (Table 1).

### 5.2. Age Group Differences and Associations

The older athletes group refer that have more parents’ support in their sports activities, (U = 1018.50; *p* = 0.006) as opposed to the younger ones (Table 2). On the other hand, the 10–12 age group state that they have already thought more about changing clubs or even quitting the sport (U = 910.50; *p* = 0.001) (Table 3).

When asked about the main reasons for choosing the club they practice basketball, the youngest mentioned the incentive from family members (42.6%) and the oldest the incentive from friends (33.3%), with the latter revealing a greater willingness to change clubs if their teammates changed as well (U = 1018.50; *p* = 0.047) (Table 3).

### 5.3. Sports Club Differences and Associations

For athletes playing in Campinho, the proximity of the sports facilities was an important factor for playing Basketball (X^2^ (1) = 3.531; *p* = 0.060) and choosing the club (X^2^ (3) = 19.84; *p* ≤ 0.001). The same happened with the Beira-Mar athletes regarding the choice of the sport (X^2^ (1) = 5.472; *p* = 0.019).

Conversely, the human relationship affects the perception the athletes from Galitos (X^2^ (1) = 2.825; *p* = 0.093) and Beira-Mar (X^2^ (1) = 4.350; *p* = 0.037) have for the sport. For them, and also for those who play for Oliveirense, the competition performance of the club influenced their choice (U = 169.50, *p* ≤ 0.001; U = 67.50, *p* = 0.001 and U = 127.50, *p* = 0.001, respectively). The former also valued the club’s conditions for playing the sport (U = 169.50; *p* ≤ 0.001). In addition, the athletes from Galitos (U = 107.50; *p* ≤ 0.001), Beira-Mar (U = 79.50; *p* = 0.004) and Oliveirense (U = 62.00; *p* ≤ 0.001) feel that their clubs have all the conditions to achieve the goals they have defined to practice the sport. Those from Galitos (U = 159.00; *p* ≤ 0.001) and Oliveirense (U = 62.00; *p* ≤ 0.001) are also satisfied with the conditions their clubs offer them. But the most satisfied with the decision they have made in choosing their club are those from Campinho (X^2^ (1) = 3.424; *p* = 0.064). Finally, Beira-Mar athletes reveal that their parents are constantly present during their activities (X^2^ (1) = 11.86; *p* = 0.003) and Oliveirense athletes consider that age (X^2^ (3) = 8.87; *p* = 0.031) and gender (X^2^ (3) = 12.95; *p* = 0.005) may influence their involvement in the practice of sports activities (Table 4 and Table 5).

### 5.4. Logistic Regression Model

The most significant factors—gender, age group, family financial conditions, friends, teammates, coach, club conditions—were included in regression models and used as predictor variables in the clubs’ sample. Table 6 shows the results of the models for each club.

The most significant predictors for choosing clubs were age group, club conditions, and teammates. The athletes who listed the most predictors, compared to the others, were those paying in Galitos. For them, these three predictors were decisive in their decision, and the last predictor is common to Campinho and Oliveirense athletes. In turn, for Beira-Mar athletes, only the age group was identified as a predictor. This means that, overall, all the predictors under analysis are important for the choice of the club as they have significance in the model. However, in an isolated way, none of them has interest analogously to the other clubs, i.e., the athletes of this club did not value the other factors.

It is worth mentioning that, although the logistic regression model concerning Oliveirense is not significant, there is a significant predictor in its choice as the club of interest. Therefore, the most valued factors are those related to the club, which indicates that personal factors are not that important.

### 5.5. Spatial Analysis

The athletes of the four clubs tend to live in the surroundings of the club’s sports facilities. Nevertheless, differences can be found between clubs. For instance, Beira-Mar athletes are the ones who live closer to the sports facilities. In turn, Campinho athletes must travel a greater distance from their households to the club’s infrastructure.

It is worth mentioning that, unlike the others, the athletes from Campinho have the least offer of sports clubs in their area of residence. The others have more club options and, despite this, most of them chose the closest club to their homes. Still, this analysis is not so straightforward for Beira-Mar, Galitos and Oliveirense clubs, as depending on where the athlete lives the difference between one club and another is not very significant.

We also found that the proximity of the sports facilities to the area of residence influenced 66.3% of the athletes (*n* = 67) in the choice of the sport modality. With respect to the choice of the sports club, for 40.6% of the youngsters (*n* = 41) the distance between the place where they live and the sports hall was an important factor when choosing the club. In contrast, for 27.8% (*n* = 28) it was not relevant and for 31.7% (*n* = 32) it was neither very/not very important. As for the way they travel to both training and playing matches, the parents/relatives play a key role in this aspect.

**Figure 2:**
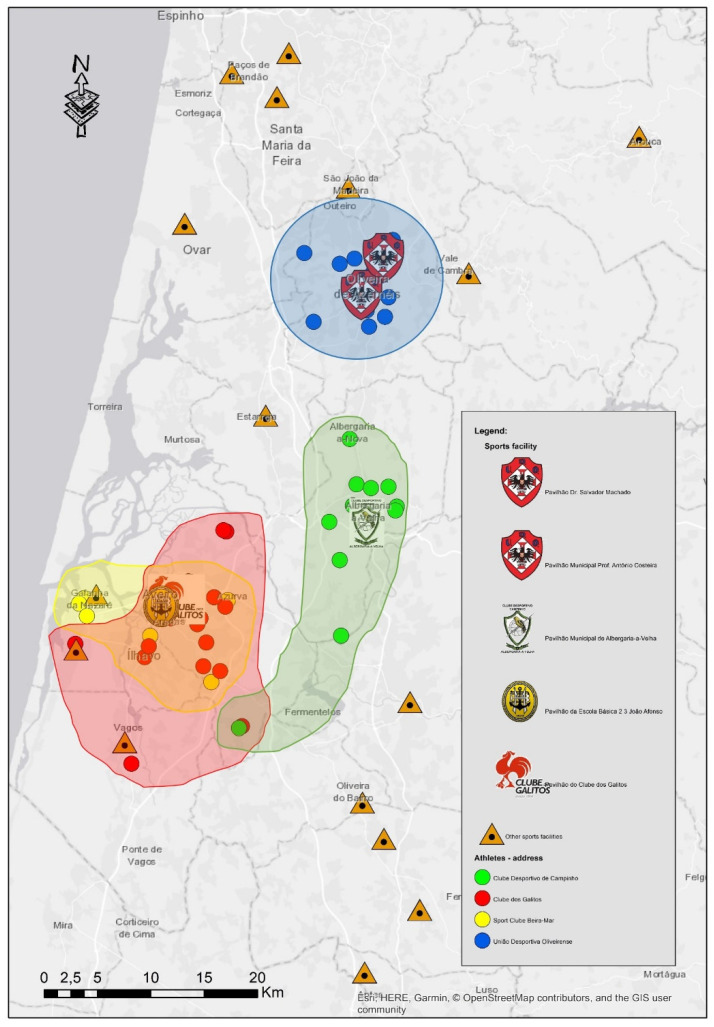
Identification of the catchment areas of the 4 clubs under study.

Still, for certain cases, the proximity of the sports facilities was not always a reason to choose the club in the place they live, having predominated other reasons to choose a club that is further away, such as having friends practicing the sport in that club. Moreover, it was possible to verify that the catchment area of Campinho and Galitos is larger than that of the other clubs (Figure 2).

### 5.6. Physical Activity/Sports during the COVID-19 Pandemic

With the suspension of sports, most athletes remained active at home (*n* = 95; 94.1%). Stull, the Galitos athletes, compared to the others, revealed a greater willingness to remain physically active (X^2^ (1) = 4.013, *p* = 0.045). Additionally, athletes in the 13–14 age group revealed that they remained more active, compared to those in the 10–12 age group (X^2^ (1) = 4.398, *p* = 0.036). Amongst Galitos and Oliveirense athletes there is a significant association between the practice of physical activity during quarantine through online sessions with teammates (X^2^ (1) = 3.397, *p* = 0.065) and in outdoor spaces suitable for sports practice (X^2^ (1) = 3.280, *p* = 0.070), respectively.

The reason for staying active varies according to age group (X^2^ (1) = 8.75, *p* = 0.034), with the youngest athletes stating the need to have moments with teammates (42.6%) and those aged 13–14 to relieve the daily stress (33.3%). The same occurs in relation to clubs, with Campinho athletes revealing the need to relieve the daily stress (X^2^ (1) = 7.488, *p* = 0.006); those from Galitos to be, virtually, with their team mates (X^2^ (1) = 3. 932, *p* = 0.047); those from Beira-Mar, the concern in maintaining physical condition to return to training in shape (X^2^ (1) = 2.751, *p* = 0.097); and, finally, those from Oliveirense, the willing they have to play the sport (X^2^ (1) = 6.231, *p* = 0.013). They felt more negative impacts on their physical condition (*n* = 49; 48.5%) and mental health (*n* = 65; 64.4%) due to the sports stoppage.

There is a significant association between gender and the reason for remaining connected to the sport, being more important for girls to maintain the connection with the sport (X^2^ (1) = 3.052, *p* = 0.081) than for boys. The same occurs in relation to age group, with older athletes showing a greater desire to return quickly to training and competition (X^2^ (1) = 7.904, *p* = 0.005). Regarding clubs, among the Campinho athletes the taste for the sport stands out (X^2^ (1) = 5.075, *p* = 0.024) and the desire to return soon to training and competition (X^2^ (1) = 4.390, *p* = 0.036). The same happened with the Oliveirense athletes, with X^2^ (1) = 5.407, *p* = 0.020 and X^2^ (1) = 8.874, *p* = 0.003, respectively. Among Galitos athletes, the concern to maintain physical fitness prevailed (X^2^ (1) = 4.812, *p* = 0.028).

More than half of the respondents (*n* = 72; 71.3%) were happy to return to training rather than afraid of contracting the virus. Sports practice in young people is essential for the acquisition of healthy habits and numerous skills, but the prolonged interruption of their path is causing a setback in their learning (*n* = 54; 53.5%), but also sadness (*n* = 37; 36.6%) and demotivation (*n* = 30; 29.7%), feelings that were almost null and void when resuming the sports practice, where contentment emerged. It was thus verified that the youngest athletes (age group 10–12 years) felt sad (X^2^ (1) = 5.732, *p* = 0.017) and the oldest (age group 13–14 years) with stress given the situation (X^2^ (1) = 2.795, *p* = 0.055). It is also important to point out that there is a significant association between the Oliveirense athletes and the feelings provoked by the absence of basketball practice, which does not occur in the other clubs, meaning that these are the ones who are more uncertain about the period they are living (X^2^ (1) = 3.256, *p* = 0.017).

## 6. Discussion

The main purpose of this research was to identify the factors that influence young athletes to choose a sports club and to understand whether the choice is related to the location of the sports facilities.

We found out a relationship between family influence, friends’ encouragement and proximity to sports facilities, extrinsic factors that contributed to the athletes’ initiation into sports, but also to their choice of sport and club. In relation to family, the results corroborate those obtained in other studies, which emphasize the existence of a positive correlation between sports participation and parents’ influence. The same happens with friends’ influence [6,34]. The study of Davidson and Jago [35], with a sample of 174 girls aged between 9 and 15 years, concluded that family support was decisive for them to start and maintain physical activity and, on the other hand, the lack/decrease of this support led them to reduce their participation and even become inactive. As for friends, the study by Torrado et al. [36] analyzing the practice of formal and informal physical activity and school sports of 783 adolescents in the 9th and 12th grades, from 16 schools in the cities of Lisbon and Setúbal, concludes that the action of friends is predominant in the initiation of sports practice and has a positive impact on both adherence and continuity of activity. According to Duncan and colleagues [34], on the other hand, parents influence the sport practice of their children at an early stage, when they are younger, and as they grow, particularly during adolescence, friends are their main source of support and persuasion. Regarding the relevance of the family for practicing sports, it is also important to refer to the support given by parents in the performance of sports activities. Older athletes, compared to younger ones, have a greater accompaniment in the performance of sporting activities. As mentioned by some authors, parents generate opportunities for participation, enroll their children in a club, provide instrumental and financial support (transportation, material), and frequently attend their activities, amongst other things [12,21,34]. The support they provide to their children is essential and, despite what would be expected, given their age, younger athletes state having a lesser accompaniment by their parents.

Regarding the proximity of sports facilities, literature documents that those two aspects may place a role with children and young individuals. For instance, the study conducted by Steinmayr and colleagues [37] for Germany showed that proximity influences female adolescents living in rural areas to join the activities and venues available for them, but on the other hand, in urban areas, no association was found between the proximity of swimming pools and tennis courts and the practice of these sports activities. Other results reported by Reimers [38] with respect to the U.S., Hong Kong, Australia and the Netherlands show that the availability of sports facilities is associated with physical activity participation in adolescents and the study by Tucker et al. [39] demonstrates that the conditions and proximity of sports facilities is a stimulant for increased physical activity, i.e., with small changes in the built environment it is possible to promote for more individuals the adoption of healthier lifestyles. Regarding our research, the results obtained do follow the same pattern of such studies. For example, the athletes from Campinho live in an area where there are no other clubs and, as such, they chose the club from their city, which is closer to them. Among the athletes from Galitos, Beira-Mar and Oliveirense this is not so evident, given the offer of other clubs in the area and also because not all of them, despite the minimal distances, chose the closest club to their home.

Research also shows that age group is a factor that has a direct influence on the choice of a club, as well as on the involvement of the practice of sports activities. This can occur because some clubs do not have enough athletes to have teams at all levels and therefore children and young people need to take this into account when choosing clubs. Teammates, who are a source of influence in various aspects of sports practice, from initiation, choice of sport and choice of club, have also been shown to be indispensable in the relationships they create among themselves to continue their sports practice. For example, Duncan et al. [34] report that there is a positive relationship between physical activity and friends, with joint participation in these activities and the support they provide each other being key in how they experience sporting experiences. The importance of friends/teammates became even more evident during confinement, with athletes mentioning that it was through online sessions with them that they stayed active at home. This indicates that teammates were an encouragement to stay active. During this period, the existence of appropriate spaces for sports relatively close to home was also important for young people to stay active, reinforcing the importance of these spaces for the population to warm up and maintain active lifestyle habits.

It is also important to mention the fact that all athletes, of both genders, before practicing this sport in their respective clubs, already knew someone who was in the club, among coaches and teammates. The relationships and interactions between these agents determine how children and young people perceive the sport, because the athletes from Clube dos Galitos and Sport Clube Beira-Mar mentioned that the relationship they have with teammates influences their perception of the sport. This is in line with the findings of the work published by Light et al. [40], focusing on swimming clubs in France, Germany and Australia, in which the importance of coaches and teammates in the sporting experiences of young people was highlighted.

Finally, the infrastructures conditions combined with team performance were factors that determined the athletes’ choice of a club over another. This was most decisive among girls and Galitos athletes. For those playing in Beira-Mar and Oliveirense, competitiveness and the results achieved by the teams were the most predominant decision factors. The only athletes who did not value these characteristics were the ones who played in Campinho, which may indicate that either they are not so concerned with competitive results or due to its location (no alternative clubs nearby) it is something that they do not value. Nevertheless, they are the only ones who feel satisfied with the decision they made in choosing their club for the sport. The importance of this factor is recognized when athletes say they are satisfied with these conditions and mention that they are meeting their goals. Similar results were found by the study conducted in Sweden by Jakobsson [41], in which adolescents, who participated in eight different sports in clubs (athletics, basketball, equestrian, soccer, floorball, handball, swimming and frisbee), not only found sports fun in terms of meaningfulness due experienced learning and development, but also found competition challenging and appealing for continuing doing sports.

## 7. Conclusions

This is one of the first studies to examine why young basketball players do sports, how they choose their team-training club and how COVID-19 has had an impact on their sports’ routine in a Southern European country. Basketball is one of the most practiced sports in Portugal [10], being Aveiro one of the regions where its practice is representative in the country. Young people in this region tend to embrace this sport, which is a critical period for development, as they initially experience the sport and progressively begin to perceive their skills and compete, which will result in the definition of certain attitudes towards the sport. These two factors differentiate this research from previous ones, in which research on Basketball athletes is scarce. Although it is between July and August that young people choose a sports club, data for this study was collected between February and April, not only due to the restrictions imposed by the pandemic, but also in order to have a clear understanding of the athletes’ choice as, by that time, they were able to make a more precise evaluation of the practice.

The influence of external (family and friends), and contextual (the material conditions and location of sports facilities) factors is quite clear in the motivation and interest to practice a sport/physical activity. The results of the questionnaire also show that the age group, teammates, and club facilities are also important to young athletes, since the last two factors have a direct influence on the practice of the sport. Athletes from Galitos and Beira-Mar, for instance, value the relationship between them and their teammates, admitting that it serves as a motivation to continue in the modality. In addition, the facilities and the competition between teams seemed important to athletes of all the clubs, except those from Campinho.

Summing up, findings show that external and contextual aspects have a big influence on kids and young individuals when it comes to choose the club, the modality and the path to a healthier lifestyle. These results are in line with the existing literature, which relates the reasons for practicing sports with certain characteristics—age, family, and friends—and seeks to understand their effect and the motivations that lead young people to practice sports, focusing mainly on modalities such as soccer and swimming [31,37,42]. Other studies also address the reasons of adherence to physical activity and sports but directed towards the intention of young people to continue in sports when they enter high school or university [43]. In this sense, although results are similar, studies focusing on the factors that lead children and young people to choose sports clubs to play basketball are scarce. They also display that, although the pandemic originated a big revolution in their life, young athletes were able to adapt to new challenges and continue practicing. Physical activity was impacted by efforts to mitigate the progression of COVID-19 infections, but widely recommended to ensure the overall well-being of individuals [17]. There were different reasons to remain active, but a common aspect amongst the athletes was the desire to remain connected to the sport, given the importance they attach to its practice, not only for the physical and mental well-being it provides, but also for the relationships created by them. Thus, the taste for sport, the need to have fun and to be virtually with teammates led them to adopt this behavior, combined with the desire to quickly resume training and competition. In short, in the face of the COVID-19 pandemic, young people’s routine changed, but the practice of sports continued to be important for them, in spite of leading to changes in the way they performed it and to some fewer positive feelings.

These findings can provide useful insights for clubs, coaches and policy makers involved in the sporting context. In fact, despite individual choices being driven by several factors, which may differ according to the context where adolescents live, hence leaving a narrow room for generalizations, with these findings local actors can become more aware of the relationship between sports practice and accessibility to sports halls, as well as the athletes’ behavior and their connection with the club and basketball practice. As such, these findings can motivate the design of initiatives and strategies to boost sports practice and to find ways for clubs to attract more adolescents.

Still, these results should be interpreted with caution due to some limitations. First, the participation of the sports clubs contacted was much lower than expected, which meant that at the end, not only we had a partial view of the regions’ sports clubs, but also the sample of athletes was small. Still, it is worth mentioning that, despite several attempts by the research team and the Basketball Association to have as many answers as possible from all clubs, the pandemic phenomenon and the adjustments that clubs have made in this context can explain such difficulty to comply with the study. A second remark relates to the fact that the two age groups surveyed are very close, which may prevent a clear perception of whether there are significant differences or associations between them.

The results obtained cannot be generalized to all clubs, age groups, and sports, due to the numerous factors that may influence young people to choose sports clubs. Therefore, future paths of research on this topic should consider a more representative sample of clubs and age groups, and even apply similar methods to other sports to understand if the factors are similar to all athletes or differ depending on the sports’ modality. From another perspective, it would also be interesting to interview club section directors to see what factors they think young people consider when choosing clubs and what persuades them to decide. On the other hand, the characteristics of the sample under study are also relevant. As previously perceived, parents can influence children’s sporting activities in various ways. According to Green and Chalip [44], when children and young people play sports for the first time, the decision to choose the sport and the place to play it, in most cases, is established by their parents. As such, future studies should include parents as participants in order to effectively understand their influence on both the sport and the club choice and, complementarily, to understand the enhancing factors and barriers that influence their behavior in following their children’s activities.

A third observation relates to the territorial component. The proximity of sports facilities was a preponderant factor in athletes’ choices and, according to Pawlowski et al. [44], the time it takes individuals to get to a sports facility has an impact on their sports’ behavior. Thus, future research should include questions concerning travel costs (time, financial, physical, etc.) from home to the sports hall. Moreover, the geographical distance should be analyzed taking different means of transport into account, such as car, public transport and bicycle.

## Figures and Tables

**Table 1:** Differences and associations at the gender level: club.

	Gender Male	Gender Female	
	*n* = 67	*n* = 34	
	Mid Value of Sample	Mid Value of Sample	U
“The distance between my household and the sports facilities was an important factor to choose the club where I play basketball”	54.69	43.72	891.50 †
“The optimal material conditions of the club infrastructures and equipment determined my choice”	44.67	63.47	715.00 **
“Factors such as the club being competitive and having district champion teams every year were important for my decision”	43.93	64.94	665.00 ***
“In this club I feel I have all the conditions to reach the goals I want to achieve as a basketball player”	45.40	62.03	764.00 **

† *p* < 0.1; ** *p* < 0.01; *** *p* < 0.001.

**Table 2:** Differences and associations at the age group level: sports practice.

	Age Group 10–12	Age Group 13–14	
	*n* = 47	*n* = 54	
	Mid Value of Sample	Mid Value of Sample	U
Do your parents usually go with you in practice and matches?	45.67	55.64	1018.50 **

** *p* < 0.01.

**Table 3:** Differences and associations at the age group level: club.

	Age Group 10–12	Age Group 13–14	
	*n* = 47	*n* = 54	
	Mid Value of Sample	Mid Value of Sample	U
Have you ever considered changing clubs or giving up the sport?	58.63	44.36	910.50 ***
If one or more teammates changed clubs, they would change with them	45.67	55.64	1018.50 *

* *p* ≤ 0.05; *** *p* < 0.001.

**Table 4:** Differences and associations at the club level: sports practice.

	Sports Club				
	Clube Desportivo do Campinho	Clube dos Galitos	Sport Clube Beira-Mar	União Desportiva Oliveirense	
	*n* = 23	*n* = 39	*n* = 15	*n* = 24	
	Mid Value of Sample	Mid Value of Sample	Mid Value of Sample	Mid Value of Sample	X^2^ (3)
Age affects the involvement in the practice of sports activities	39.13	52.82	46.67	61.13	8.87 *
Gender is a factor that influences adherence to sports activities	37.26	53.73	44.57	63.75	12.95 **

* *p* ≤ 0.05; ** *p* < 0.01.

**Table 5:** Differences and associations at the club level: club.

	Sports Club				
	Clube Desportivo do Campinho	Clube dos Galitos	Sport Clube Beira-Mar	União Desportiva Oliveirense	
	*n* = 23	*n* = 39	*n* = 15	*n* = 24	
	Mid Value of SAMPLE	Mid Value of Sample	Mid Value of Sample	Mid Value of SAMPLE	X^2^ (3)
“The distance between my home area and the sports facilities was an important factor when I was choosing the club where I play basketball”	70.17	43.09	35.37	55.25	19.84 ***
“The optimal material conditions of the club infrastructures and equipment determined my choice ”	31.70	62.74	43.47	55.13	19.14 ***
“In choosing the club, I considered factors such as the club being competitive and having district champion teams every year ”	27.85	61.91	49.63	56.31	21.94 ***
“In this club I feel I have all the conditions to reach the goals I want to achieve as a basketball player”	22.83	61.21	45.20	63.42	37.11 ***
Level of satisfaction with club conditions	31.76	65.81	35.07	55.33	31.68 ***

*** *p* < 0.001.

**Table 6:** Logistic regression model.

	**Sport Clube Beira-Mar**			
	***B***	**SE**	**Wald**	**95% CI**
**Model**				
Constant	−2.488	0.788	9.979	
Age group	1.553	0.759	4.186 *	[1.067, 20.903]
Sex	−1.85	0.7	0.069	[0.211, 3.281]
Family financial conditions	0.595	0.685	0.755	[0.474, 6.934]
Friends	0.19	0.847	0.05	[0.230, 6.358]
Teammates	−19.825	8585.282	0	[0.000]
Coach	−1.392	1.158	1.446	[0.026, 2.405]
Club conditions	0.066	0.932	0.005	[0.172, 6.638]
Model Summary	χ^2^ (6) = 0.140 *, *R*^2^ Nagelkerke = 0.247			
Classification Accuracy (%)	85.1			
	**União Desportiva Oliveirense**			
	***B***	**SE**	***t***	**95% CI**
**Model**				
Constant	−1.268	0.552	5.28	
Age group	0.37	0.535	0.479	[0.507, 4.133]
Sex	−0.001	0.515	0	[0.365, 2.740]
Family financial conditions	−0.076	0.571	0.018	[0.302, 2.840]
Friends	0.841	0.611	1.894	[0.700, 7.689]
Teammates	0.08	0.645	0.016	[0.306, 3.838]
Coach	−0.031	0.796	0.001	[0.204, 4.614]
Club conditions	−2.454	1.121	4.794 *	[0.010, 0.773]
Model Summary	χ^2^ (6) = 0.095, *R*^2^ Nagelkerke = 0.143			
Classification Accuracy (%)	76.2			
	**Clube Desportivo do Campinho**			
	***B***	**SE**	**Wald**	**95% CI**
**Model**				
Constant	−1.719	0.638	7.267	
Age group	−248	0.691	0.128	[0.201, 3.027]
Sex	−1.778	0.1.158	2.358	[0.017, 1.635]
Family financial conditions	−0.167	0.747	0.05	[0.273, 5.113]
Friends	−1.337	0.87	2.359	[0.048, 1.446]
Teammates	−0.829	0.947	0.765	[0.068, 2.795]
Coach	−0.597	0.887	0.453	[0.319, 10.323]
Club conditions	−3.608	0.861	17.541 ***	[6.819, 199.669]
Model Summary	χ^2^ (6) = 0.375 ***, *R*^2^ Nagelkerke = 0.570			
Classification Accuracy (%)	77.2			
	**Clube dos Galitos**			
	***B***	**SE**	**Wald**	**95% CI**
**Model**				
Constant	0.036	0.475	0.006	
Age group	−0.952	0.493	3.729 *	[0.147, 1.014]
Sex	0.582	0.49	1.414	[0.686, 4.674]
Family financial conditions	0.237	0.535	0.197	[0.276, 2.250]
Friends	−0.367	0.593	0.383	[0.217, 2.214]
Teammates	1.313	0.634	4.282 *	[1.072, 12.880]
Coach	0.275	0.733	0.141	[0.313, 5.542]
Club conditions	−2.379	0.896	7.048 **	[0.016, 0.536]
Model Summary	χ^2^ (6) = 0.204 **, *R*^2^ Nagelkerke = 0.277			
Classification Accuracy (%)	61.4			

Note: SE = standard error; CI = confidence intervals; * *p* ≤ 0.05; ** *p* < 0.01; *** *p* < 0.001.

## Data Availability

Not applicable.
